# Tele-monitored tDCS rehabilitation: feasibility, challenges and future perspectives in Parkinson’s disease

**DOI:** 10.1186/s12984-019-0481-4

**Published:** 2019-01-31

**Authors:** Alberto Cucca, Kush Sharma, Shashank Agarwal, Andrew Seth Feigin, Milton Cesar Biagioni

**Affiliations:** 1 The Marlene and Paolo Fresco Institute for Parkinson’s and Movement Disorders, Department of Neurology, NYU School of Medicine, 222 East 41st Street, New York, NY 10017 USA; 2 Department of Neurology, NYU School of Medicine, New York, NY USA

**Keywords:** Telemedicine, Transcranial direct current stimulation, Neuro-rehabilitation, Parkinson’s disease

## Abstract

Transcranial direct current stimulation (tDCS) is a modality of non-invasive brain stimulation involving the application of low amplitude direct current via surface electrodes on the scalp. tDCS has been studied in healthy populations and in multiple brain disorders and has the potential to be a treatment for several neuropsychiatric conditions by virtue of its capability of influencing cognitive, motor and behavioral processes. tDCS is a generally safe technique when performed within standardized protocols in research or clinical settings. Furthermore, tDCS portability, high acceptability and user-friendly interface makes it highly appealing for telemedicine practices. The term “telemedicine” refers to the procedures, educational strategies, and care services that are remotely administered by means of different communication technologies, with the final goal of increasing access to care for individuals and for improving public health. The use of telemedicine combined with tDCS protocols is increasing, although the safety of this approach in different clinical settings awaits further assessment. While “do-it-yourself” tDCS should be discouraged due to the unknown risk of adverse events, the implementation of tele-monitored tDCS (tele-tDCS) within standardized frameworks ensuring safety, tolerability, and reproducibility may allow this technology to reach larger clinical populations and bypass some of the common barriers preventing access to health services and clinical trials. This review will discuss the current evidence supporting the feasibility of tele-tDCS paradigms and their therapeutic potential, with particular emphasis on the implications for patients with Parkinson’s disease.

## Introduction

The past decade of this century has been marked by the steady development of new technologies that are now able to connect broad sectors of populations to medical providers and health care institutions. The term “telemedicine” (i.e. healing at a distance) encompasses a broad range of telecommunication technologies that allow for health services to be delivered remotely [1]. These services include, among others, the administration of diagnostic assessments, clinical monitoring, prevention strategies, therapeutic supervision, education, consultation and psychological support [2]. The obvious advantage of telemedicine is the possibility of circumventing logistic barriers of face-to-face health services by increasing accessibility for people with disability and for those living in remote geographic areas with poor access to healthcare facilities [3].

There are further potential advantages related to the implementation of telemedicine protocols. These include the possibility of providing real-time monitoring of adverse events, facilitating therapeutic compliance, reducing social disparities in the access to care, promoting patients’ active role in diagnostic and therapeutic processes, and reducing waiting time and economic burden, particularly for people affected by chronic illnesses [4, 5]. Eventually, the applications of telemedicine may involve monitoring multiple symptoms remotely by means of portable devices, with relevant therapeutic implications, especially for clinically fragile populations [6].

On the other hand, the rapid development of telemedicine poses radical new challenges. The intensive exchange of medical information through different platforms carries potential risks related to loss of confidentiality on large volumes of sensitive data. These risks must be minimized by implementing appropriate data protection strategies [7]. Further, while telemedicine may potentially allow access to larger populations, including individuals eligible for research trials, the prerequisite of a subject’s ability to use telecommunications may introduce a systematic selection bias leading to the exclusion of those sectors of the population with poor or no technological skills. Although age and familiarity with technology do not necessarily influence the access to telemedicine, it has been shown that exposure to telemedicine practices does correlate with the level of technological confidence [8].

Nevertheless, in the past years, the availability of technology-enabled communications has grown dramatically and their cost/effectiveness has improved accordingly. As a result, the remote administration of diagnostic and therapeutic procedures has become a widely consolidated clinical practice in different medical fields, including urgent care, robotic surgery, imaging and physical rehabilitation [9–12]. Recently, our center has completed an interdisciplinary and home-based model for a population with Parkinson’s Disease (PD) [13]. In these patients, access to specialized care has been shown to decline dramatically as the disease progresses, mainly due to cumulative physical disability compounded by several psychosocial factors [14]. Indeed, the lack of access to specialized care delivered by appropriately trained physicians has been linked to increased morbidity, mortality and healthcare costs [13].

Transcranial direct current stimulation (tDCS) is an investigational device broadly used in experimental and clinical neuroscience with a wide range of potential therapeutic applications. To date, tDCS has been applied for the study and treatment of several conditions by virtue of its capability of influencing cognitive, motor and behavioral processes related to the pathophysiology of different brain disorders [15]. One of the most significant advantages of tDCS over other methods of non-invasive brain stimulation (NIBS) is its portability, low cost and safety [16]. Furthermore, due to its favorable tolerability and easier applicability, tDCS could be easily incorporated into telemedicine-based protocols by means of specialized devices that are designed for tele-monitored tDCS (tele-tDCS) [17]. The development of tele-tDCS interventions in clinical research holds great potential by removing accessibility barriers, supporting a greater number of subjects in research studies and enabling the possibility to introduce patients largely underrepresented in research, including those burdened by greater morbidity or by being homebound. Lastly, the rigorous administration of tele-tDCS research protocols involving customized devices and headgear may improve the rate of recruitment, reduce attrition, relieve patients’ burden and increase cost/efficacy while maintaining clinical trial standards.

The aim of this narrative review is to present the reader with the latest evidence supporting the feasibility of tele-tDCS paradigms and the potential near future applications of this technique for both experimental and clinical purposes. Finally, the specific challenges and theoretical implications for patients affected by PD will be critically discussed.

## Telemedicine-based tDCS protocols

### tDCS, essentials

tDCS is a modality of NIBS involving the application of a low amplitude direct current (DC) via surface electrodes on the scalp for a predetermined time in a relatively safe manner. In tDCS, the cerebral cortex is stimulated through a continuous, weak current (usually 1 to 2.5 mA) which alters brain function by changing the neuronal resting membrane potential to either cause depolarization (under the anode) or hyperpolarization (under the cathode) [18]. The principal mechanism of action of tDCS is a subthreshold modulation of neuronal membrane potentials, thus modulating spontaneous neuronal firing activity depending on the previous physiological state of the brain target area [19, 20]. Additional mechanisms of action include the possibility to harness neuroplasticity through long-term potentiation (LTP) and long-term depression mechanisms (LTD) as well as to modify functional connectivity throughout distributed cortico-subcortical networks, etc. [21, 22]. Although the exact mechanisms underlying tDCS effects are not fully known at a molecular level, growing evidence suggests non-linear effects mediated, at least partially, by localized shifts of intracellular Ca2+ concentration [23, 24].

Growing evidence accumulated through randomized controlled trials (RCT) supports the potential of tDCS for the treatment of various disorders, such as chronic pain, fatigue, cognitive abnormalities, substance-related disorders, and depression [25]. Many of these therapeutic areas are relevant to patients burdened by chronic neurological diseases, physical disability, or those that are homebound. The safety of this technique has been addressed and tested by multiple researchers who have concluded that tDCS, as applied and monitored in compliance to the international safety guidelines, is a safe and well-tolerated intervention [26]. However, the safety and tolerability of tDCS on more vulnerable populations and tele-tDCS paradigms remain to be fully elucidated.

### Rationale and pre-requisites for tele-monitored tDCS

A major limitation to the extensive clinical application of NIBS protocols has been that most research studies have involved small sample sizes and short duration, resulting in trials without proper power analysis and predefined intention to treat designs. The large majority of available trials has indeed involved a limited number of sessions and participants as both clinicians and investigators face challenges in the recruitment and retention of participants in large-scale NIBS trials [27–30]. From the patient’s perspective, the need to undergo multiple, consecutive sessions spanning weeks or months can be particularly cumbersome, especially in presence of highly disabling symptoms [19]. Adapting tDCS technology for an easy at-home use while meeting rigorous standards of experimental reproducibility and safety monitoring could make this intervention more suitable for larger research studies involving a broader patient population.

**Fig. 1:**
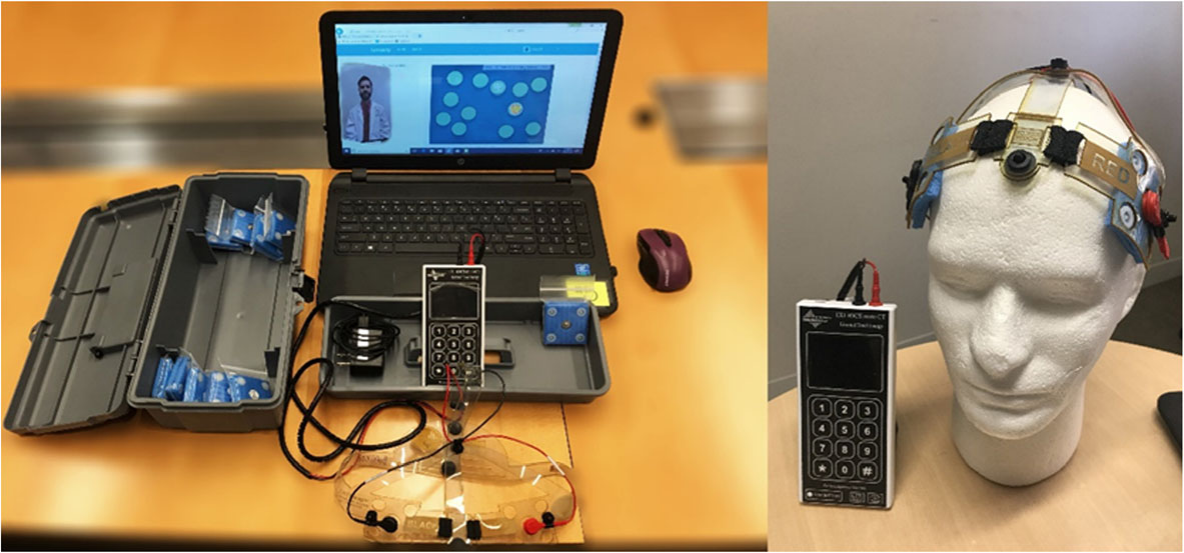
Example of tele-monitored tDCS (tele-tDCS) setup including the tDCS device (for at-home sessions), head strap and the videoconferencing platform. The depicted tDCS device is a Soterix Mini-CT tDCS. The device delivers direct electrical current through saline-soaked sponge electrodes (5 cm × 5 cm) snapped to a custom-made head strap. The head strap has clear labeling for reliable electrode placement (right picture). This configuration provided a uniform bi-hemispheric dorsolateral prefrontal cortex montage centered using a nasion marker. The supervising study technician is shown in the laptop screen (left picture) as a study participant would see it during video-conferencing

One prerequisite to tele-tDCS is the availability of equipment specifically designed to allow for remote use and a customized headset for easy and reliable placement of the electrodes. Simply utilizing the same devices designed to be operated by health professionals may compromise subject safety and affect experimental reproducibility since inter-individual differences in technological skills and environments may not be adequately addressed [31]. Customized devices for remote usage should include clear instructions regarding the operation of the device and the adequate setup of headset and electrodes montage [32] (Table 1). The headgear should be designed to allow simple and consistent placement of the electrode at the desired location, thus facilitating reliable setup (for example, the headset can be labeled in different colors to confirm that cables and electrodes are properly matched). Special markers should be used to ensure that the headset fits reliably on subject’s head, while friendly usable (size-fitted) head straps or caps should be provided to hold the sponge electrodes in a still position throughout the duration of each session. In Fig. 1 we describe a typical experimental tele-tDCS device designed for remote use in all its components.

**Table 1:** Steps, challenges, and solutions for daily remotely supervised tDCS (RS-tDCS) sessions. Modified from Riggs et al. with authors’ permission [33]

Step	Challenge	Solution
1. Having all supplies available and ready	Losing or misplacing Supplies	Bountiful supply of pads with proper package of the tool box
2. Connecting to the internet	A stable internet connection	The Wi-Fi password readily available and troubleshooting any problems being faced
3. Starting video conference with remote control	Providing the study personnel with the required password	Phone call beforehand for the required password for remote connection
4. Attaching electrodes	Improper attachment without alignment	Each step clearly illustrated in the patient instructional video with remote supervising
5. Proper placement of head strap	Improper location	
6. Preparing the device	Not clear identification of each keypad button with each step	
7. Checking contact quality	Understanding contact quality grade	Lay language words, correcting any issues with study personnel for improving contact quality
8. Starting the stimulation	Using the correct start code provided by study personnel	Lay language terms and instructional video on how to start stimulation
9. Computer-based cognitive training	Understanding and learning each game	Lay Language and positive reinforcement for each game played
10. Ending the stimulation	Hearing the beeping sound	Informing patient the session is over
11. Clean up of the electrodes	Proper cleaning of electrodes to avoid corrosion	Teaching the patient on how and where to clean the saline solution
12. Charging battery	Charging battery as needed	Step by step manner in instructional videos on how to charge the device

In addition to dedicated devices, tele-tDCS should employ an adequate level of control by specifically trained research staff or expert clinical professionals. On this topic, “do it yourself” (DIY) tDCS practices should be highly discouraged in light of the inherent risks involving subject’s safety and experimental reproducibility [33]. Visual confirmation obtained via HIPAA-compliant videoconferencing can be used to ensure proper set up and control for contact quality while meeting optimal standards of data handling and confidentiality. Dedicated training via instruction manual and demonstration video should also be considered to further maximize the chances of optimal electrode placement.

### Experimental challenges with tele-monitored tDCS trials

The biological effects of tDCS, as any other NIBS technique, are essentially determined by two factors: extrinsic (related to the intervention) and intrinsic (related to the stimulated subject) [34]. Extrinsic factors are related to the amount of energy, the pattern of current flow and the number on sessions delivered to the brain. In tDCS, these include the electrode characteristics, the technical preparation and the device-controlled voltage waveform [35]. However, for the same dose of energy delivered, different intrinsic factors of the subject contribute to the individuals’ biological outcome, including the pharmacological profile, age, gender, genetic characteristics, brain state, the subject’s circadian rhythm, etc. [36].

In general, the best way to ensure consistent and reliable electrode placement is to implement video-supervised sessions during which the user, with or without caregivers, are followed in real time by study personnel until electrodes are properly placed and adequate contact is confirmed. Indeed, tele-tDCS protocols have consistently showed optimal adherence, high tolerability and uniformity across sessions, while self-administered tDCS without any degree of supervision has been associated with a high dropout rate [37, 38].

As far as the device-controlled voltage waveform is involved, the so-called “hardware approach” involves the administration of a pre-determined voltage by programmed devices operating for a limited number of sessions. After completion of the last session, the device no longer provides any output. Alternatively, in the so-called “software approach”, each tDCS session is enabled with a pre-set duration and intensity by a specific code provided to the user and/or caregiver by the research staff. After a single session, the device remains inactive until a new code is provided to unlock the next session [39].

**Fig. 2:**
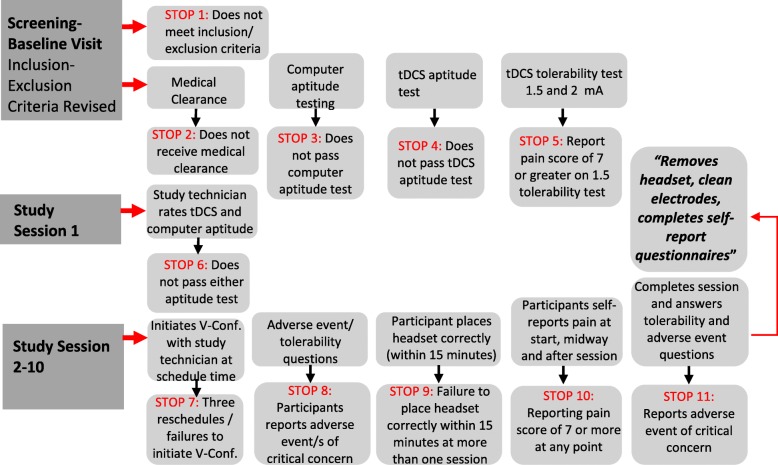
Algorithm diagram with stop criteria for tele-monitored tDCS. Modified version from Kasschau et al. with authors’ permission. This protocol included user and/or caregiver capability to participate in tDCS, check list of procedures for safe placement of electrodes and head strap, dose control, monitoring of compliance and adverse events, and clear guidelines for discontinuation of sessions and/or study participation. Note various stop criteria determining when a subject is no longer able to safely participate in the study

In 2015, Charvet et al. proposed a standardized framework for trials utilizing a tele-tDCS protocol defined as “*remotely-supervised tDCS*” (RS-tDCS) in order to ensure the same level of uniformity, compliance and reproducibility that would be expected by tDCS sessions administered in the clinic [39]. This protocol included dedicated training of staff, user and/or caregiver, assessment of the user’s capability to participate in tDCS remotely, checklist of simple procedures for safe placement of electrodes and headgear, strict dose control, monitoring of compliance and adverse events, and clear guidelines for discontinuation of sessions and/or study participation. In our studies, we utilize a modified version of the proposed workflow algorithm by Kasschau, Charvet and colleagues that is summarized in Fig. 2 [40].

Shaw et al. analyzed the feasibility and tolerability of a remotely supervised protocol involving anodal tDCS over left dorsolateral prefrontal cortex (DLPFC) paired with computer-based cognitive training [41]. The authors pooled the available evidence from three distinct patient populations: 26 participants with multiple sclerosis (MS) undergoing an open-label study, 20 MS participants in a randomized, double-blind, sham-controlled trial, and six patients with PD who were recruited. A total of 748 RS sessions were successfully completed in approximately 1 year, with adverse events not exceeding well-known mild side effects of transient burning sensations of skin, tingling, or itching being the most commonly reported. Overall, these findings supported the feasibility and safety of RS-tDCS as well as its potential generalizability to different patient populations. However, it should be noted that the feasibility of different and/or more complex montages, for example high definition tDCS, remains to be determined.

While protocols involving the remote use of tDCS should be structured enough to ensure reproducibility and safety, certain population-specific considerations should also be made when applying tele-tDCS to patients with different clinical profiles. Charvet et al. provided some examples of these population-specific adaptations, where peculiar issues encountered while delivering RS-tDCS in people affected by attention-deficit/hyperactivity disorder, depression, multiple sclerosis, and severe chronic illnesses requiring palliative care were critically discussed [39]. It is likely that the capability of a subject who was initially deemed eligible to undergo tele-tDCS may fluctuate over time in relation to his/her cognitive, behavioral and physical symptoms. This is particularly relevant for patients suffering from chronic mood disorders or cognitive abnormalities. In patients with PD, a peculiar challenge to tele-tDCS may be posed by the presence of motor and non-motor fluctuations. Overall, population-specific considerations should always be incorporated into tele-tDCS designs, for example by including a flexible involvement of subjects’ caregivers and/or an adaptive monitoring of potential technical issues encountered during trial conduct.

### Feasibility of tele-monitored tDCS for neurologic disorders

The available evidence supporting the telemedicine-based use of tDCS remains limited to small exploratory trials showing significant heterogeneity in terms of patient populations, primary outcome, and experimental design. In 2018, Palm et al. conducted a systematic review including available protocols and original research involving the home use of tele-tDCS for the treatment of various neuropsychiatric disorders [38]. From five RCT, three open label studies and four case reports the overall rate of side effects did not exceed those commonly observed with conventional tDCS in lab settings operating within the international safety lines. Regular visits and remote conferences by means of different communication technologies seemed critical in ensuring the correct performance of stimulation and minimizing attrition. The blinding integrity of controlled studies appeared to be optimal, with no significant differences in guesses for active or sham treatments. Palm et al. evaluated the overall experimental quality of the RCT determined by adherence to the proposed protocol, technical quality of stimulation, including electrode placement and dose delivery, safety monitoring and adverse events handling, modality of data storage, strategies to foster adherence and capture meaningful clinical changes. Out of the included RCT trials, only one fulfilled all of the above parameters. More specifically, this RCT of André et al., reported specific improvements in executive functions in 21 patients affected by vascular dementia undergoing four sessions of anodal at-home tDCS over the right DLPFC [42].

Furthermore, Palm et al. proposed a new nomenclature to highlight the methodological differences between different uses of tele-tDCS. More specifically, the authors proposed a distinction between a “*domiciliary use*” of tDCS for compassionate or interventional purposes, in which stimulation parameters are advised by the medical staff but remain entirely dependent on patient’s compliance, vs a “*remotely supervised use*”, in which the patient or the caregiver activates a preprogrammed device secured against manipulation. In remotely supervised protocols, supervision is offered by means of different technological platforms (telephone, videoconferencing, email, smartphone applications, etc.) in order to overcome potential technical difficulties and ensure optimal adherence. A third identified setting is the “*remotely controlled tDCS*”, in which the device, while still operated by patients or caregivers, remains under constant remote control of the medical staff. Regardless of its practical implications, this nomenclature reflects the need to refine the terminology used in the various trials to foster a greater experimental uniformity and facilitate the systematic analysis of the available data. Additionally, while the evidence supporting the safety and feasibility of tele-tDCS is gradually emerging, the efficacy of this new modality of neuromodulation remains to be validated though well-powered, well designed studies.

## Tele-monitored tDCS in Parkinson’s disease

### Telemedicine and movement disorders

Neurodegenerative diseases, particularly movement disorders, are known to be characterized by chronic, progressive disability and reduced mobility. These conditions are frequently compounded by a number of psychosocial factors that can further reduce both accessibility and adherence to medical care. Furthermore, in certain geographical and socioeconomic backgrounds, the effective care of movement disorders is hindered by the scarcity of specifically trained health care professionals as well as by limited access to dedicated facilities [43].

PD is the second most common neurodegenerative disorder after Alzheimer’s Dementia. Given the current demographic trend, the global burden of PD will increase rapidly, particularly in those developing economies where access to care is more limited [44]. It was recently observed that the majority of individuals with PD have a very limited access to care, with more than 40% of patients lacking appropriate neurologic care in the United States [45]. Even among individuals initially receiving dedicated and/or interdisciplinary medical attention the cumulative disability related to the disease progression may eventually lead to a homebound status that compromises access to specialized care. Furthermore, PD patients may be more likely to be lost to follow-up because of common comorbidities such as fatigue, mood disorders, apathy, and anxiety as well as by the onset of complications related to the prolonged pharmacological treatment such as motor fluctuations and dyskinesia [46]. The use of telemedicine to offer “virtual house calls” for PD has been suggested to be comparable to in-office visits as it is feasible, cost-effective, and acceptable to patients [47, 48]. The potential pool of patients that may benefit from telemedicine is therefore remarkable. In addition to an improved access to specialized care, telemedicine-based protocols may foster participation in clinical trials and provide meaningful observational data on the natural course of disease in the most advanced stages [49]. Without effectively reaching those patients burdened by higher disability and clinical severity, it is difficult to carry out inclusive research protocols and validate optimal therapies [50].

### Tele-monitored tDCS evidence in Parkinson’s disease

We reviewed the state of research on tele-tDCS applied to patients affected by PD. The research included the following databases: Pubmed, Google Scholar, Scopus, Semantic Scholar, Clinical Trials.gov and Research Gate. The terms “remotely-supervised”, “at-home”, “domiciliary”, “tele-monitored”, “telehealth” and “telemedicine” were searched in cross-combination with “Parkinson’s Disease” and “transcranial direct current stimulation”. We found three studies regarding the use of telemedicine-based tDCS in this specific population.

Shaw and colleagues published the first feasibility and safety trial with a paradigm of remotely supervised tDCS (RS-tDCS) that included six PD patients in 2017. The study reported feasibility and safety of RS-tDCS over 10 daily sessions paired with computer-based cognitive training in 52 patients. Of the 60 RS-tDCS sessions in PD participants, side effects did not differ from commonly reported side effects of conventional tDCS [41].

The authors of the present review have recently completed an exploratory, open label study of remotely supervised anodal tDCS over the left DLPFC paired with cognitive training in PD participants [51]. Based on the methods of Shaw et al., each participant completed 10 daily RS-tDCS sessions (20-min, 1.5–2.0-mA, bi-hemispheric DLPFC montage, left anodal), over a span of 2 weeks. The study enrolled 16 PD participants with moderate disease severity. One participant terminated early from the study due to exertional angina that resolved after a stenting procedure; this was deemed unrelated to the study. A total of 152 RS-tDCS sessions were completed during the study, with 100% compliance and only mild adverse events providing evidence in favor of the feasibility and safety of tele-rehabilitation in PD participants. In terms of clinical outcomes, a small but significant improvement in both motor scores and total scores of the Unified Parkinson’s Disease Rating Scale was observed (a widely used scale for quantification of PD cumulative disability). Within motor scores, the greatest improvement was in axial/balance symptoms. Importantly, axial/balance symptoms including gait failure are often unresponsive to optimal pharmacological treatments and are known to be poor prognostic factors in PD. Agarwal et al. acknowledged several study limitations inherent to the open-label, exploratory design, which does not allow controlling for placebo effect. Further limitations include the small sample size and the absence of a long-term follow-up assessment. However, the results of this study provided further evidence supporting the feasibility and safe therapeutic application of tele-tDCS in patients PD.

After the successful completion of the first open label exploratory study, our group is conducting an ongoing double-blind, pilot, RCT testing the effects of RS-tDCS using a DLPFC montage to ameliorate fatigue and cognitive slowing in PD (https://clinicaltrials.gov/show/NCT03189472). The protocol involves 10 daily sessions of bi-frontal 20-min RS-tDCS (2 mA, F3-F4 montage, left anodal) followed by an optional open label phase consisting of 10 additional sessions. Seventeen participants completed 330 tele-tDCS sessions (170 double blind and 160 open label). Preliminary feasibility and safety results showed no serious adverse events, only mild to moderate side effects and 100% compliance. All participants but one opted to undergo the open label phase [52].

**Fig. 3:**
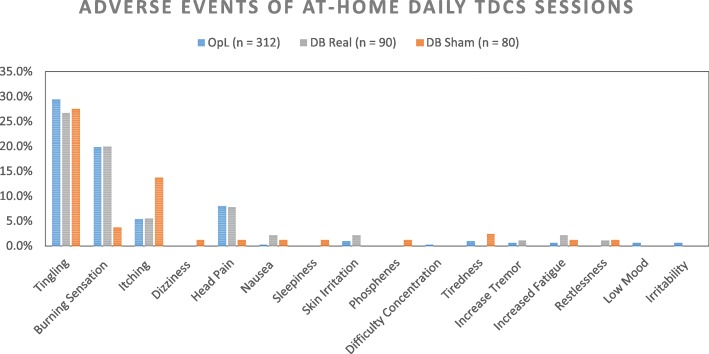
Pooled data of frequency of adverse events experienced with remotely supervised tDCS (RS-tDCS) under real time video-conferencing in Parkinson’s disease patients. Presented here, a total of 312 sessions for open label (OpL), 90 sessions for double blind real (DB), and 80 sessions for DB sham. During sham tDCS, patients received only 60 s of stimulation at the beginning and at the end of the 20 min tDCS sessions

Pooling the available safety data in PD from both studies together, totaling 482 tele-tDCS sessions, the side effects and adverse events were similar than previously published studies performing conventional tDCS in lab settings (Fig. 3), with the exception of a single severe adverse event which was deemed unrelated to study procedures. Based on these preliminary results, at-home RS-tDCS therapy seems acceptable and well tolerated in this population, with the advantages of ease of recruitment and subject retention. Data regarding the efficacy of this technology to ameliorate fatigue and cognitive slowing in PD is still pending upon completion of the study.

### Specific challenges inherent to Parkinson’s disease population

Telemedicine protocols may be intimidating to those who are not familiar with the technology, and cognitive impairment is a common finding in advanced PD. Participation of such patients has been shown to be increased by the availability of caregivers agreeable to be trained in the use of the equipment and assist the patient during sessions. In selected populations (e.g. limited mobility, co-morbid dementia), the support of a caregiver, spouse or significant other might be important for tele-tDCS paradigms. Increased caregiver’s burden is a well-documented occurrence in PD [53]. This factor must be considered in the implementation of tele-tDCS protocols, as additional responsibilities may further challenge caregivers’ strain.

Fluctuating motor performance, severe and/or unpredictable OFF states, severe freezing and bothersome dyskinesia or tremor could also be present temporarily in a given patient. Special precautions (e.g. providing video-conferencing platforms to detect these states) and schedule flexibility to conduct sessions might improve compliance, safety and tolerability in these particular cases.

Finally, from an experimental viewpoint, patient’s pharmacological state while receiving tDCS neuromodulation can affect the brain activation state and connectivity by modulating neuronal propensity to fire and undergo plastic phenomena. In patients with PD, this is particularly noteworthy, as changes in cortical excitability and neuroplasticity are critically influenced by dopamine bioavailability, and the institution of a dopaminergic therapy can influence the subsequent neurophysiologic and behavioral effects of stimulation [54]. The potential influence of subject’s contingent dopaminergic state should be carefully considered when implementing experimental tDCS protocols and a rigorous control of patient’s pharmacological state, particularly in subjects experiencing motor fluctuations, should be always pursued.

## Future perspectives

The broad application of non-invasive brain stimulation techniques, including tDCS, is currently limited by different factors. First, the body of the available evidence still rests on small-sized studies carried out with exploratory designs. As such, these studies are known to be particularly prone to the risk of type I and type II statistical errors. A second order of limitation is posed by the high heterogeneity of stimulation parameters and methods between the published trials. These differences result in a limited comparability between the various experimental protocols. Finally, a third important constrain is the paucity of studies with multiple tDCS sessions (i.e. beyond 10 sessions) as the current evidence suggests higher chances of harnessing cumulative biological effects following multiple sessions of stimulation over time. The development of tele-tDCS paradigms may specifically address some of these challenges improving protocol standardization and adherence while minimizing attrition. This may be particularly noteworthy in patients burdened by chronic motor disability or living in remote geographic areas. In the near future, the broad availability of different communication technologies may favor the implementation of new personalized models of care in neurology and rehabilitation. In this setting, the therapeutic value and overall safety of tele-tDCS remain to be determined through appropriately designed trials. The current limited evidence suggests high acceptance rate and overall optimal feasibility but further studies are needed to corroborate these preliminary findings.

## Conclusions

tDCS is a relatively safe and tolerable non-invasive neuro-modulation technique that could be incorporated into telemedicine protocols in light of its portability and easy operability. The use of tele-tDCS within standardized frameworks ensuring safety, tolerability, and reproducibility in adequately selected patients may expand access to care and allow for the inclusion of larger populations into clinical trials while minimizing attrition and improving cost/effectiveness. Most patients affected by PD worldwide face multiple barriers preventing a consistent access to specialized, effective, and interdisciplinary care. Current evidence supports feasibility and safety of tele-tDCS protocols in the setting of remotely supervised videoconference sessions delivered to patients affected by PD. In these patients, specific challenges to the extensive implementation of tele-tDCS include the possibility of motor and non-motor fluctuations, cognitive deficits, polypharmacy, psychiatric comorbidities and risk of caregiver’s burden. Although the safety and feasibility of these protocols in PD patients await further validation, preliminary data seem to suggest optimal adherence and acceptability in this particular patient population opening the field for larger research initiatives needed to define the therapeutic potential of this intervention.

## Abbreviations

NIBS: Non-invasive brain stimulation
PD: Parkinson’s disease
RCT: Randomized controlled trial
RS: Remotely supervised
tDCS: Transcranial direct current stimulation
tele-tDCS: tele-monitored tDCS
